# Sex‐ and age‐specific effect of known type 2 diabetes mellitus on incident mild cognitive impairment five years later: Results from the population‐based Heinz Nixdorf Recall study

**DOI:** 10.1002/dad2.70130

**Published:** 2025-06-11

**Authors:** Anna Lena Platzbecker, Janine Gronewold, Sara Schramm, Susanne Moebus, Andreas Stang, Börge Schmidt, Christian Weimar, Martha Jokisch, the Heinz Nixdorf Recall Study Investigative Group

**Affiliations:** ^1^ Department of Neurology and Center for Translational Neuro‐ and Behavioral Sciences (C‐TNBS) University Hospital of Essen University of Duisburg‐Essen Essen Germany; ^2^ Fliedner University of Applied Science Düsseldorf Düsseldorf Germany; ^3^ Institute for Medical Informatics, Biometry, and Epidemiology University Hospital Essen University of Duisburg‐Essen Essen Germany; ^4^ Institute of Urban Public Health University Hospital of Essen University of Duisburg‐Essen Essen Germany; ^5^ BDH‐Klinik Elzach gGmbH Elzach Germany

**Keywords:** cardiovascular risk factor, cognitive decline, epidemiology, mild cognitive impairment, prevention, type 2 diabetes mellitus

## Abstract

**Introduction:**

As studies on the association between type 2 diabetes mellitus (T2DM) and mild cognitive impairment (MCI), including amnestic (aMCI) and non‐amnestic (naMCI) subtypes, vary by sex and age, we investigated the sex‐ and age‐specific effects of T2DM on incident MCI after five years in a population‐based sample.

**Methods:**

A total of 145 participants with T2DM and 1322 without T2DM were included. MCI was defined using established criteria excluding subjective cognitive decline. Adjusted relative risks (aRRs) were calculated considering age, education, body mass index, smoking, and alcohol intake, and stratified by sex and age (middle‐aged: 50–65 years; old‐aged: 66–80 years).

**Results:**

MCI occurred in 39.3% (*n* = 57) of participants with T2DM versus 27.5% (*n* = 363) without (aRR: 1.29, 95% confidence interval [CI]: 0.97–1.73). Middle‐aged men showed an association with naMCI (aRR: 2.35, 95% CI: 1.26–4.39) and middle‐aged women with aMCI (aRR: 2.05, 95% CI: 0.58–7.21).

**Discussion:**

T2DM increases MCI risk, particularly in middle‐aged individuals with poorly controlled T2DM, emphasizing the need for prevention strategies.

**Highlights:**

- Longitudinal results from the population‐based Heinz Nixdorf Recall study in Germany.
- Incident mild cognitie impairment (MCI) was more common in type 2 diabetes mellitus (T2DM; 39% vs 28%).
- T2DM affects incident MCI and subtypes in middle‐aged, not old‐aged; stronger in men with poorly controlled T2DM.
- Importance of enhancing age‐ and sex‐specific prevention at the population level.

## 1 BACKGROUND

In 2020, ≈13% of the global population, or 1 billion individuals, were 60 years of age and older.^1^, ^2^ By 2050, this demographic is projected to more than double, driven by increases in life expectancy and an aging global population. As a result, late‐life cognitive decline and dementia will become increasingly prevalent, with an estimated 152 million people expected to be affected by dementia by 2050.^2^, ^3^ This growing trend poses significant challenges for health care systems and social services, as well as a profound human crisis, impacting not only the quality of life for those affected but also placing an emotional and physical burden on families and caregivers. The socioeconomic burden of dementia will be substantial, making it an essential task to address this issue.

Type 2 diabetes mellitus (T2DM) is also a fast‐growing health problem. Approximately one‐third of people 65 years of age and older have known or unknown T2DM.^4^ Patients with T2DM are, furthermore, at higher risk of increased cognitive decline.^5^ Moreover, T2DM is linked to a 60% increased risk of dementia and cognitive impairment.^6^, ^7^

Mild cognitive impairment (MCI) is considered a transitional stage between the cognitive changes of normal aging and cognitive impairment that interferes with everyday activities representing an early stage of dementia.^8^ There are two subtypes of MCI: amnestic MCI (aMCI; or MCI due to Alzheimer's disease [AD])^9^ and non‐amnestic MCI (naMCI). In aMCI, there is objective evidence of impairment in one or more cognitive domains, including memory. If objective evidence of impairment is found predominantly in cognitive domains other than memory, such as attention, visuoconstruction, or executive function (naMCI), it is more likely that the impairment is due to vascular or other non‐AD pathology. Annual conversion rates from MCI to AD range from 10.2% to 33.6% (median 19%^10^). There are limited data on conversion rates from naMCI to dementia. However, the risk of vascular dementia (VaD) 3.8 years later was significantly higher in MCI patients with more subcortical hyperintensities at baseline.^11^ The presence of T2DM seems to elevate the risk of this progression.^12^

The impact of T2DM on cognitive function appears to differ between men and women, but the specifics are unclear.^13^, ^14^ Women generally maintain better midlife cognition than men (mean age = 68 years^15^); however, cognitive decline with age is more pronounced in women, independent of diabetes status.^16^ Winkler et al.^17^ analyzed the available data of this study sample cross‐sectionally and found an association between known T2DM and MCI for the middle‐aged group (50–65 years). The association was stronger with aMCI in women and with naMCI in men. To determine whether the same pattern exists over time and to explore the implications for prevention, we now examine the data longitudinally to assess the temporal relationship between T2DM and cognitive impairment.

The aim of the present study was to investigate the sex‐specific association between T2DM and incident MCI and MCI subtypes five years later in an initially cognitively healthy middle‐aged and old‐aged population‐based study sample.

## 2 METHODS

### 2.1 Study participants

Participants from the prospective population‐based Heinz Nixdorf Recall (Risk Factors, Evaluation of Coronary Calcification, and Lifestyle) study were selected randomly from mandatory city registries in the Ruhr area, Germany, and were invited to partake in the study, as reported previously.^18^, ^19^ For a detailed description of the study design, see Schmermund et al.^18^ A total of 4814 participants, 45–75 years of age, were enrolled in the initial examination (t0) between 2000 and 2003, and were scheduled for follow‐up assessments every 5 years (t1, *n *= 4157, 2005–2008; t2, *n *= 3087, 2010–2015; median follow‐up time between t0 and t2: 10 years; median follow‐up time between t1 and t2: 5 years). The data collection process involved standardized interviews, clinical examinations, comprehensive laboratory tests, and self‐administered questionnaires (see Section 2.3). A standardized cognitive performance assessment was introduced at t1 and expanded to include three further tests at t2 (see Section 2.2). Alongside the in‐person follow‐up examinations at the study center, participants were sent annual postal follow‐up questionnaires.

**FIGURE 1:**
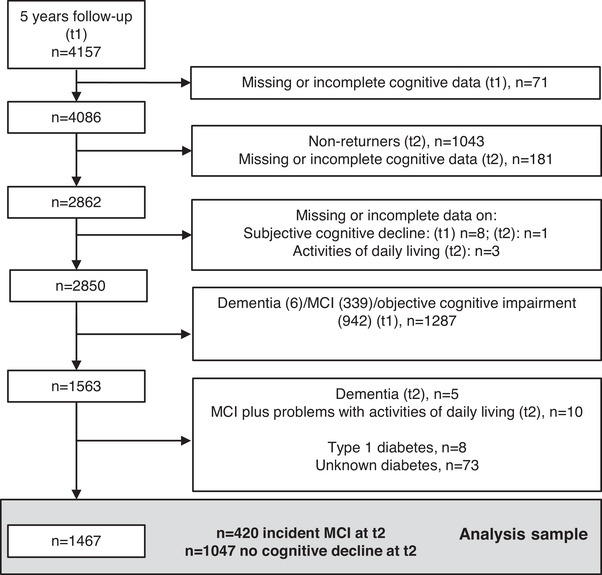
Flowchart for the present study. Abbreviations: MCI, mild cognitive impairment following previously published criteria excluding subjective cognitive decline; t1, first follow‐up examination (2005–2008), t2, second follow‐up examination (2010–2015).

Figure 1 shows a flowchart of participants included in this analysis starting at t1 (*n *= 4157), when cognitive assessment was implemented. Due to missing or incomplete cognitive data at t1, 71 participants were excluded. Of the remaining 4086 participants, 1043 did not show up at t2 for different reasons, and 181 participants had missing or incomplete cognitive data at t2 resulting in 2862 participants. Twelve participants were excluded due to missing or incomplete data on subjective cognitive decline and activities of daily living. As we have included only cognitively unimpaired participants at t1, we excluded 942 participants with objective impairment not meeting the previously published MCI (no subjective cognitive complaint present), 339 participants with MCI, and 6 demented participants at t1. Of the remaining 1563 participants, 5 participants were demented at t2 and 10 participants had MCI plus problems with activities of daily living and therefore did not fulfill the MCI or dementia diagnosis (see Section 2.2.2). Eight participants had type 1 diabetes mellitus at t1, and 73 participants had unknown diabetes at t1. Thus, 1467 participants (420 with incident MCI at t2 and 1047 with no MCI at t2) were included in the analyses.

The study was approved by the institutional review board of the University Duisburg‐Essen and followed established guidelines of good epidemiological practice. Written informed consent was provided by all participants.

### 2.2 Assessment of cognitive status

Briefly, at t2, the cognitive performance assessment consisted of eight tests and covered the following four cognitive domains: (1) attention: Trail Making Test A, and Color‐word test card 1 and card 2; (2) executive function: Trail Making Test B, Labyrinth test, Color‐word test interference performance, and verbal fluency; (3) verbal memory: eight word list immediate and delayed recall; (4) visuoconstruction: clock‐drawing test. At t1, Trail Making Test A and B and the Color‐word test were not yet assessed. Detailed descriptions of the assessment procedures and domain construction has been reported earlier.^20^, ^21^ Cognitive impairment was defined present if at least one domain score (attention, executive function, verbal memory) was more than 1 SD below the age‐ and education‐adjusted mean or a score of 3 or higher in visuoconstruction.^20^, ^22^

**RESEARCH IN CONTEXT**

1. **Systematic review**: The authors reviewed the literature using PubMed and meeting abstracts and presentations. Although type 2 diabetes mellitus (T2DM) is an established risk factor for the development of dementia, studies on the longitudinal association of mild cognitive impairment (MCI) and its subtypes with T2DM are inconsistent depending on the age and sex groups studied. Relevant citations are appropriately cited.
2. **Interpretation**: The aim of the present study was therefore to investigate the sex‐specific association between T2DM and incident MCI and MCI subtypes five years later in an initially cognitively healthy middle‐aged and old‐aged population‐based study sample.
3. **Future directions**: Our longitudinal data confirm T2DM as a risk factor for MCI, especially in middle‐aged participants with poorly controlled T2DM. These results underscore the importance of enhancing tailored prevention efforts at the population level, not only for T2DM but for mitigating the risk of MCI and preserving cognitive performance.

#### 2.2.1 Cognitive diagnoses

The MCI diagnosis was based on meeting the first three of four or all of the following published MCI criteria^23^: (1) cognitive impairment in at least one of the above reported four domains; (2) normal functional abilities in daily activities; (3) no dementia diagnosis; (4) subjective cognitive decline (SCD) assessed with the question “In comparison to 2 years ago would you rate your memory function as better, the same, or worse?” (SCD was defined as present if the participant's answer was “worse”). When applying all four MCI criteria at t2, only 137 incident MCI cases were identified, of which 15 had T2DM, making sex and age‐stratified analyses statistically uncertain. Thus, we decided to use criterion 1–3 for MCI diagnosis (without the necessity of SCD). However, we performed a sensitivity analysis for the total sample, with incident MCI with criterion 1–4 (*n *= 137) stratified solely by sex.

To examine incident MCI and MCI subtypes at t2 in cognitively healthy participants at t1, participants with MCI (meeting criteria 1–3 or 1–4) at t1 were excluded as detailed above (Figure 1). Participants at t2 not meeting MCI criteria as detailed above were categorized as “no MCI.”

Dementia diagnosis was established by either a physician's prior diagnosis of dementia in accordance with the fourth edition of the Diagnostic and Statistical Manual of Mental Disorders (DSM‐IV) criteria or the administration of cholinesterase inhibitors (coded as N06DA in the WHO's Anatomical Therapeutic Chemical [ATC] classification) or other anti‐dementia medications (coded as N06DX^24^).

### 2.3 Assessment of diabetic status at t1

Known T2DM at t1 was defined as either a self‐reported diagnosis from a physician or the use of anti‐diabetic medication. Participants with diabetes were inquired about the type of diabetes. All participants were asked to fast a minimum of 8 h before the examination, with 79% of the study participants adhering to this fasting requirement.^17^ Unknown T2DM at t1 was defined as the absence of a self‐reported diagnosis of T2DM, along with a fasting glucose level of ≥126 mg/dL (≥7 mmol/L) or a random blood glucose level of ≥198 mg/dL (≥11 mmol/L;^25^). Hemoglobin A1c (HbA1c) was measured using immunonephelometry at 340/700 nm (BNII nephelometer, Dade‐Behring, Deerfield, IL, USA). All analyses were done in the central laboratory of the University Hospital of Essen.

### 2.4 Assessment of covariates

Computer‐assisted interviews at t1 and t2 collected information about medical/family history (e.g., coronary heart disease (CHD), smoking, alcohol consumption, and socioeconomic status).^18^, ^19^ Medical examinations included blood and urine sampling, blood pressure measurement, and the estimated glomerular filtration rate (eGFR) using the Modification of Diet in Renal Disease (MDRD) formula. Stroke events were validated by an independent committee. Genotyping was performed using Cardio‐MetaboChip BeadArrays to identify apolipoprotein E (*APOE*) ε2, ε3, and ε4 alleles. Participants underwent three seated blood pressure measurements with an automated oscillometric blood pressure device (Omron, HEM‐705CP) and classified according to JNC‐7 (Joint National Committee on Prevention, Detection, Evaluation, and Treatment of High Blood Pressure^26^; systolic blood pressure and/or diastolic blood pressure: normal (<120 mmHg and <80 mmHg), pre‐hypertension (120–139 mmHg or 80–89 mmHg), stage 1 (140–159 mmHg or 90–99 mmHg), and stage 2 (≥160 mmHg or ≥100 mmHg, or intake of antihypertensive medication)). Depressive symptoms were assessed using the German version of the Center for Epidemiologic Studies Depression scale (CES‐D) short form.^27^ Information on educational attainment (defined by combining school and vocational training as total years of formal education according to the International Standard Classification of Education) was stratified into four categories: ≤ 10 years; 11–13 years; 14–17 years; ≥ 18 years. Standardized measured height and weight were used for calculating body mass index (BMI; kg/m^2^). “Current smoking” was defined as a history of cigarette smoking during the past year. If there was a history of smoking (longer than one year ago), participants were defined as “former smoker.” Participants were asked about the frequency and quantity of alcohol intake, which was converted into gram alcohol per day (g/d).

The variables used for model adjustment were selected using a directed acyclic graph (DAG) based on the current literature on the topic (see Figure S1). The DAG was constructed using the online software DAGity v2.3.^28^ The minimally sufficient adjustment set included the following variables: age, sex (only for the total sample), education, BMI, smoking status, and alcohol intake. Based on this framework, hypertension, depression, CHD, *APOE* ε4, stroke, and kidney function (eGFR) were not included in the main adjusted models.

### 2.5 Statistical analyses

To compare participants stratified by sex and age group (middle‐age: 50–65 years; old‐age: 66–80 years) with incident MCI versus no MCI at t2 regarding sociodemographic and clinical characteristics, we performed Mann–Whitney *U* tests for continuous variables and Pearson *χ*2 tests for categorical variables. We used a log‐linear regression model with a Poisson estimate and robust variance to obtain relative risks (RRs) and corresponding 95% confidence intervals (95% CIs).

The outcome variables were incident MCI and MCI subtypes; the exposure variable was known T2DM (vs no T2DM) at t1. To include the available information on the duration of T2DM, we expanded the analyses to include T2DM (vs no T2DM) at t0 as the exposure variable. Because cognition was not assessed at t0, we also utilized data from cognitively healthy participants at t1 for the analyses with T2DM at t0 as the exposure variable. We calculated two models: first, a crude model with only the exposure as an independent variable; second, a fully adjusted model including all variables suggested by the DAG.^28^

## 3 RESULTS

Demographic and clinical characteristics of all study participants, stratified by sex, age‐group (middle‐age: 50–65 years, old‐age: 66–80 years), and MCI status at t2, are depicted in Table 1. In total, 145 (10%) participants had known T2DM at t1. Of these, 52 (36%) were newly diagnosed cases within the last 5 years. Thirty (21%) had an undiagnosed T2DM at t0, and 63 (43%) already had a known T2DM diagnosis at t0. Men demonstrated higher eGFR values and more elevated glucose levels than women, although there was no significant difference in HbA1c values between sexes. A larger proportion of middle‐aged men surpassed the HbA1c cutoff (see Table 1).

**TABLE 1:** Sociodemographic and clinical characteristics assessed at t1 of male and female participants (*n* = 1467) of the Heinz Nixdorf Recall study, Germany, 2005–2008 (t1) and 2010–2015 (t2).

	Men (*n *= 670)				Women (*n *= 797)			
	Middle‐age (50–65 years)		Old‐age (66–80 years)		Middle‐age (50–65 years)		Old‐age (66–80 years)	
	ø incident MCI at t2 (*n *= 311)	Incident MCI at t2 (*n *= 110)	ø incident MCI at t2 (*n* = 157)	Incident MCI at t2 (*n *= 92)	ø incident MCI at t2 (*n *= 398)	Incident MCI at t2 (*n *= 120)	ø incident MCI at t2 (*n *= 181)	Incident MCI at t2 (*n *= 98)
Age, mean ± SD	58.50 ± 4.11	58.32 ± 4.34	70.26 ± 3.57	71.10 ± 3.48	58.17 ± 4.01	58.21 ± 4.13	70.50 ± 3.54	71.10 ± 3.86
*Education*								
≤10 years, *n* (%)	11 (4)	7 (6)	6 (4)	3 (3)	12 (3)	6 (5)	41 (22.7)	20 (21)
11–13 years, *n* (%)	114 (37)	45 (41)	65 (42)	38 (41)	252 (63)	78 (65)	120 (66.3)	66 (67)
14–17 years, *n* (%)	98 (31)	38 (35)	54 (34)	40 (44)	58 (15)	24 (20)	14 (7.7)	7 (7)
≥18 years, *n* (%)	88 (28)	20 (18)	32 (20)	11 (12)	76 (19)	12 (10)	6 (3.3)	5 (5)
BMI, kg/m^2^, mean ± SD ^a^	27.9 ± 3.9	28.3 ± 3.8	27.6 ± 3.6	28.3 ± 4.1	27.0 ± 5.1	27.2 ± 5.3	28.2 ± 4.7	28.8 ± 4.9
*Smoking*								
Never smoked, *n* (%)	86 (28)	37 (34)	55 (35)	36 (39)	167 (42)	52 (43)	126 (70)	72 (74)
Former smoker, *n* (%)	166 (53)	45 (41)	91 (58)	45 (49)	149 (37)	44 (37)	43 (24)	21 (21)
Current smoker, *n* (%)	58 (19)	28 (25)	11 (7)	11 (12)	82 (21)	24 (20)	12 (6)	5 (5)
Alcohol, g/day, mean ± SD ^b^	18.3 ± 19.5	14.4 ± 19.6	14.2 ± 15.3	13.1 ± 17.7	6.6 ± 10.5	4.6 ± 7.1	4.7 ± 9.0	4.3 ± 8.5
Known T2DM, *n* (%)	22 (7.1)	18 (16.4)	27 (17.2)	19 (20.7)	17 (4.3)	7 (5.8)	22 (12.2)	13 (13.3)
Glucose, mg/dL, mean ± SD ^c^	108.35 ± 18.94	114.49 ± 28.52	112.10 ± 16.97	115.25 ± 23.26	102.10 ± 11.88	103.63 ± 13.59	106.16 ± 16.15	107.66 ± 15.42
HbA1c, %, mean ± SD ^d^	5.50 ± 0.56	5.67 ± 0.65	5.61 ± 0.63	5.73 ± 0.64	5.46 ± 0.47	5.48 ± 0.44	5.60 ± 0.66	5.67 ± 0.50
HbA1c ≥ 6,5%, *n* (%) ^d^	15 (5)	13 (12)	10 (6)	9 (10)	8 (2)	3 (3)	7 (4)	7 (7)
History of stroke, *n* (%)	7 (2.3)	3 (2.7)	3 (1.9)	6 (6.5)	8 (2)	3 (2.5)	3 (1.7)	5 (5.1)
eGFR, mL/min/1,73 m^2^, mean ± SD ^e^	70.1 (9.3)	71.9 (9.9)	67.0 (10.4)	64.4 (13.4)	64.1 (9.7)	64.2 (8.5)	60.8 (8.8)	61.2 (9.1)

Abbreviations: BMI, body mass index (kg/m^2^); eGFR, estimated glomerular filtration rate; HbA1c, hemoglobin A1c; MCI, mild cognitive impairment defined following previously published criteria excluding subjective cognitive decline; SD, standard deviation; t1, first follow‐up (2005–2008); t2, second follow‐up (2010–2015); T2DM, type 2 diabetes mellitus.

^a^ Missing values for *n *= 5 participants.

^b^ Missing values for *n *= 11 participants.

^c^ Missing values for *n *= 9 participants.

^d^ Missing values for *n *= 22 participants.

^e^ missing values for *n *= 218 participants.

At t2, 57 (39.3%) participants with T2DM versus 363 (27.5%) without T2DM met criteria for incident MCI. For participants with T2DM we found a RR of 1.43 (95% CI: 1.08–1.89) for incident MCI compared to participants without T2DM (adjusted RR: 1.31, 95% CI: 0.98–1.74; see Table 2; all following reported RR and 95% CI in the results section are fully adjusted). In stratified analyses, we found a 1.86 (95% CI: 1.10–3.14) fold increased RR for incident MCI for T2DM in middle‐aged men.

**TABLE 2:** Effect of known T2DM at t1 on incident MCI and MCI subtypes at t2 for the total sample and stratified by age‐group and sex, Heinz Nixdorf Recall study, Germany, 2005–2008 (t1) and 2010–2015 (t2).

		MCI cases *n* (%)		Unadjusted		Adjusted^*^	
	Ø T2DM	Ø T2DM	T2DM	RR (95% CI)	*p*‐value	RR (95% CI)	*p*‐value
				1.0 reference		1.0 reference	
Total sample	*MCI*	363 (27)	57 (36)	1.43 (1.08–1.89)	.012	1.29 (0.97–1.73)	0.083
	*aMCI*	105 (8)	18 (12)	1.56 (0.95–2.58)	.080	1.34 (0.80–2.25)	0.261
	*naMCI*	258 (20)	39 (27)	1.38 (0.98–1.93)	.062	1.31 (0.93–1.86)	0.128
Middle‐age (50–65 years)	*MCI*						
	Total	205 (23)	25 (39)	1.67 (1.10–2.53)	.016	1.66 (1.08–2.56)	0.020
	Men	92 (24)	18 (45)	1.87 (1.13–3.09)	.016	1.86 (1.10–3.14)	0.020
	Women	113 (23)	7 (29)	1.28 (0.59–2.74)	.533	1.22 (0.55–2.67)	0.626
	*aMCI*						
	Total	61 (7)	8 (13)	1.79 (0.86–3.75)	.120	1.55 (0.72–3.32)	0.265
	Men	34 (9)	5 (13)	1.40 (0.55–3.58)	.482	1.16 (0.44–3.05)	0.762
	Women	27 (5)	3 (13)	2.29 (0.69–7.54)	.174	2.05 (0.58–7.21)	0.265
	*naMCI*						
	Total	144 (16)	17 (27)	1.61 (0.98–2.67)	.062	1.71 (1.02–2.88)	0.042
	Men	58 (15)	13 (33)	2.14 (1.17–3.90)	.013	2.35 (1.26–4.39)	0.007
	Women	86 (17)	4 (17)	0.96 (0.35–2.61)	.932	0.94 (0.34–2.62)	0.902
Old‐age (66–80 years)	*MCI*						
	Total	158 (35)	32 (39)	1.12 (0.76–1.63)	.566	1.11 (0.75–1.64)	0.595
	Men	73 (36)	19 (41)	1.15 (0.69–1.90)	.591	1.11 (0.65–1.91)	0.704
	Women	85 (35)	13 (37)	1.07 (0.60–1.91)	.830	1.09 (0.60–1.99)	0.771
	*aMCI*						
	Total	44 (10)	10 (12)	1.25 (0.63–2.49)	.518	1.34 (0.66–2.72)	0.412
	Men	26 (13)	6 (13)	1.02 (0.42–2.74)	.968	1.08 (0.43–2.76)	0.867
	Women	18 (7)	4 (11)	1.55 (0.52–4.58)	.428	1.49 (0.49–4.53)	0.485
	*naMCI*						
	Total	114 (26)	22 (27)	1.07 (0.68–1.68)	.787	1.02 (0.65–1.65)	0.899
	Men	47 (23)	13 (28)	1.22 (0.66–2.26)	.525	1.12 (0.57–2.17)	0.745
	Women	67 (27)	9 (26)	0.94 (0.47–1.88)	.853	0.97 (0.47–1.97)	0.922

*Note*: Data are from log‐linear regression models with Poisson estimates and robust variance.

Abbreviations: CI, confidence interval; MCI, mild cognitive impairment following previously published criteria excluding subjective cognitive decline; øT2DM, no type 2 diabetes mellitus; RR, relative risk; T2DM, known type 2 diabetes mellitus.

* Adjusted for age, sex (only for the total samples), education (≤10 years, 11–13 years, 14–17 years, ≥18 years), BMI (body mass index [kg/m^2^]), alcohol intake (pure alcohol [g/day]), smoking status (current smoker, former smoker, never smoked).

Considering MCI subtypes in the total cohort, regression analyses revealed a RR of 1.34 (95% CI: 0.80–2.25) for incident aMCI for T2DM and a RR of 1.31 (95% CI: 0.93–1.86) for incident naMCI for T2DM. Stratified analyses revealed both associations to be strongest in the middle‐aged population (aMCI: 1.55, 95% CI: 0.72–3.32; naMCI: 1.71, 95% CI: 1.02–2.88). Regarding incident aMCI, the observed trend was predominantly driven by female participants (middle‐aged: 2.05, 95% CI: 0.58–7.21; old‐aged: 1.49, 95% CI: 0.49–4.53). For naMCI the association was strongest in middle‐aged men (RR: 2.35, 95% CI: 1.26–4.39).

Regression analyses with T2DM (vs no T2DM) at t0 as exposure produced similar results. These regression analyses also revealed an elevated RR for incident MCI for T2DM at t0 in the total cohort (RR: 1.41; 95% CI: 1.03–1.91; see Table 3). In stratified analyses, the association was evident in middle‐aged men (RR: 1.78, 95% CI: 1.05–3.02).

**TABLE 3:** Effect of both known and unknown T2DM at t0 on incident MCI and MCI subtypes at t2 for the total sample and stratified by age‐group and sex, Heinz Nixdorf Recall study, Germany, 2000–2003 (t0) and 2010–2015 (t2).

		MCI cases *n* (%)		Unadjusted		Adjusted^b^	
	Ø T2DM	Ø T2DM	T2DM	RR (95% CI)	*p*‐value	RR (95% CI)	*p*‐value
				1.0 reference		1.0 reference	
Total sample	*MCI*	371 (27)	49 (43)	1.55 (1.15–2.09)	0.004	1.41 (1.03–1.91)	0.030
	*aMCI*	110 (8)	13 (11)	1.56 (0.95–2.58)	0.080	1.34 (0.80–2.25)	0.261
	*naMCI*	261 (19)	36 (31)	1.62 (1.14–2.30)	0.007	1.59 (1.10–2.24)	0.014
Middle–age (50–65 years at t1)^a^	*MCI*						
	Total	207 (23)	23 (40)	1.71 (1.12–2.65)	0.014	1.66 (1.07–2.60)	0.024
	Men	93 (24)	17 (43)	1.74 (1.04–2.92)	0.036	1.78 (1.05–3.02)	0.031
	Women	114 (23)	6 (35)	1.55 (0.68–3.53)	0.295	1.40 (0.60–3.25)	0.436
	*aMCI*						
	Total	64 (7)	5 (9)	1.21 (0.49–3.00)	0.683	1.08 (0.42–2.72)	0.873
	Men	36 (9)	3 (8)	0.79 (0.24–2.57)	0.701	0.73 (0.22–2.40)	0.601
	Women	28 (6)	2 (12)	2.11 (0.50–8.84)	0.309	1.54 (0.35–6.72)	0.331
	*naMCI*						
	Total	143 (16)	18 (32)	1.95 (1.19–3.18)	0.008	2.07 (1.25–3.42)	0.004
	Men	57 (15)	14 (35)	2.34 (1.30–4.20)	0.004	2.54 (1.40–4.61)	0.002
	Women	86 (17)	4 (23)	1.37 (0.50–3.74)	0.538	1.34 (0.48–3.74)	0.579
Old–age (66–80 years at t1)^a^	*MCI*						
	Total	164 (35)	26 (45)	1.29 (0.85–1.94)	0.235	1.22 (0.79–1.89)	0.366
	Men	76 (36)	16 (44)	1.17 (0.68–2.00)	0.570	1.12 (0.63–1.99)	0.702
	Women	88 (34)	10 (50)	1.47 (0.77–2.83)	0.247	1.43 (0.72–2.84)	0.312
	*aMCI*						
	Total	46 (10)	8 (14)	1.41 (0.67–2.99)	0.370	1.42 (0.65–3.10)	0.383
	Men	27 (13)	5 (13)	1.03 (0.40–2.67)	0.954	1.01 (0.41–3.00)	0.847
	Women	19 (7)	3 (15)	2.05 (0.61–6.92)	0.250	1.69 (0.47–6.14)	0.424
	*naMCI*						
	Total	118 (25)	18 (58)	1.24 (0.75–2.03)	0.402	1.17 (0.70–1.96)	0.554
	Men	49 (23)	11 (29)	1.25 (0.65–2.40)	0.509	1.13 (0.56–2.28)	0.744
	Women	69 (27)	7 (35)	1.31 (0.60–2.86)	0.491	1.32 (0.59–3.00)	0.500

*Note*: Data are from log‐linear regression models with Poisson estimates and robust variance.

Abbreviations: CI, confidence interval; MCI, mild cognitive impairment following previously published criteria excluding subjective cognitive decline; øT2DM, no type 2 diabetes mellitus; RR, relative risk; T2DM, known type 2 diabetes mellitus.

^a^ Participants were aged 45‐60 and 61‐75 years at T2DM diagnosis at t0.

^b^ Adjusted for age, sex (only for the total samples), education (≤10 years, 11–13 years, 14–17 years, ≥18 years), BMI (body mass index [kg/m^2^]), alcohol intake (pure alcohol [g/day]), and smoking status (current smoker, former smoker, never smoked)

Considering MCI subtypes in the total cohort, regression analyses revealed an elevated RR for incident aMCI for T2DM at t0 (RR: 1.34, 95% CI: 0.80–2.25) and for incident naMCI for T2DM at t0 (RR: 1.59, 95% CI: 1.10–2.24). Parallel to the results mentioned above, we found an association of incident aMCI and T2DM in female participants (middle‐aged: RR: 1.54, 95% CI: 0.35–6.72; old‐aged: RR: 1.69, 95% CI: 0.47–6.14). Regarding incident naMCI, we found particularly increased RRs in the middle‐aged population (RR: 2.07, 95% CI: 1.25–3.42), and especially for middle‐aged men (RR: 2.54 95% CI: 1.40–4.61).

The sensitivity analysis, incorporating all four MCI criteria confirmed this pattern and showed robust results for men (RR: 1.40, 95% CI: 0.73–2.70), but a diminished effect for women (RR: 0.48, 95% CI: 0.15–1.54). We were unable to stratify by age group due to small sample sizes.

## 4 DISCUSSION

The main finding of this population‐based longitudinal study is an effect of T2DM on incident MCI and incident MCI subtypes in initially cognitively healthy, middle‐aged but not old‐aged participants. This association is strongest in men, who also exhibited the poorest glycemic control. Men and women exhibit differences in health behavior, with women often taking more responsibility for their health.^29^ Tailoring preventive strategies to address these sex‐specific disparities is crucial, not only for conditions like T2DM but also for mitigating risks of MCI.

The etiology of dementia in people with T2DM is multifactorial and not fully understood. Preclinical studies suggest various mechanisms such as neuronal insulin resistance, impaired insulin signaling, inflammation, mitochondrial dysfunction, and vascular damage, which increase deposition of amyloid beta, tau proteins, and glycogen synthase kinase 3 beta (GSK3β), leading to an earlier onset of dementia in individuals with an impaired glucose metabolism.^30^ Although the association between T2DM and cerebrovascular changes resulting in VaD (20% of all forms of dementia) seems more obvious, studies also show the association of T2DM with neurodegenerative forms of dementia, in particular with AD as the most common form (70% of dementia cases^31^, ^32^). There is good evidence on the association between T2DM and incident VaD and AD showing an ≈2.5‐ fold and 1.5‐ fold increased risk, respectively.^33^ T2DM significantly increases the risk of VaD due to the chronic vascular damage caused by elevated blood sugar levels and furthermore leads to atherosclerosis, microvascular damage, and inflammation, all of which impair cerebral blood flow and contribute to cognitive decline associated with VaD.^34^ Furthermore, T2DM is closely linked to an increased risk of AD due to insulin resistance, chronic inflammation, and oxidative stress. These factors contribute to the accumulation of amyloid plaques and tau tangles in the brain, impairing cognitive function and accelerating neurodegeneration, which are hallmarks of AD.^35^, ^36^

In contrast to VaD, pathological alterations associated with AD emerge up to 20 years before the initiation of cognitive decline.^37^, ^38^ In terms of prevention for either form of dementia, identifying and treating modifiable risk factors, for example T2DM, while individuals are cognitively unimpaired is of major importance.^23^, ^39^, ^40^, ^41^

Previous studies have identified associations between T2DM, MCI, its subtypes, and dementia, with these relationships varying based on the age and sex of the individuals studied. Consistent with the cross‐sectional findings of Winkler et al.^17^ (2014), our longitudinal data revealed a similar association between T2DM and both MCI and naMCI in middle‐aged men. Our data replicate a higher incidence of MCI among men.^42^ We also identified an association between T2DM and aMCI in women, although the strength of this association was weaker compared to previous studies.^6^, ^17^ This is in line with Moran et al.,^15^ finding better cognitive health in women independent of diabetic status. In our study, middle‐aged women exhibited lower glucose levels and were less likely to have an HbA1c score ≥6.5% compared to middle‐aged men, suggesting a less advanced stage of pathophysiology. This highlights once again that the severity of poorly controlled T2DM may increase MCI risk, which is biologically plausible given the damaging effects of chronic hyperglycemia on cognitive function.

Furthermore, unlike Roberts et al.,^42^ we did not find an association of T2DM and MCI and MCI subtypes in the old‐aged population. However, our study population was younger (70.6 years at t1 [+ 5 years at t2] vs 79.3 years) and had a lower prevalence of known T2DM, with fewer individuals experiencing severe complications or advanced disease.

The duration of T2DM has been reported to affect the risk of developing dementia and may also be linked to MCI.^43^ Our data revealed even stronger associations between T2DM and both MCI and naMCI in the middle‐aged cohort, particularly among middle‐aged men, when considering T2DM at t0 (10 years prior to MCI diagnosis; 45‐60 years old at T2DM diagnosis at t0) as the exposure. These findings highlight the importance of lifestyle modifications and vascular risk factor management for preventive strategies.^44^ Because T2DM is considered a midlife risk factor for dementia, early, adequate diagnoses and treatment is of major importance.^41^ Furthermore, diabetes treatments, such as metformin, sodium‐glucose cotransporter 2 (SGLT2) inhibitors, and glucagon‐like peptide‐1 (GLP‐1) receptor agonists, may play a role in the prevention of T2DM. The latter, an incretin hormone released in the lower digestive tract following nutrient intake, significantly lowers plasma glucose and enhances glycemic control in patients with T2DM when activated by receptor agonist drugs. In addition, patients with T2DM who are treated with a GLP‐1 receptor agonist were less likely to report cognitive impairment or dementia.^45^, ^46^ Furthermore, magnetic resonance imaging (MRI) scans revealed a slower decrease in brain volume in individuals with mild to moderate AD who were treated with GLP‐1 receptor agonist, but the exact implications of these effects require further research.^47^ For individuals with T2DM, who have MCI, guidelines advise reducing medications that carry a risk of hypoglycemia.^43^ Previously published guidelines recommend screenings for cognitive impairment in adults with T2DM who are age 65 and older.^43^

In addition, our findings emphasize the necessity of preventing T2DM by managing cardiovascular risk factors, promoting a healthy lifestyle, conducting routine preventive examinations, and implementing regular glucose screening to reduce the risk of cognitive impairment. As dementia progresses to more advanced stages, diabetes‐specific treatments are unlikely to significantly slow further cognitive decline.^44^ At this time point, cognitive decline can also lead to impaired diabetes management, which highlights the importance of maintaining cognitive health, especially for patients with T2DM.

There are limitations to our study. As is the case with all longitudinal studies, selective attrition may have biased our results, as non‐returners and excluded participants showed less favorable cardiovascular and cognitive profiles at t1 (see Table S1). This is partly due to our inclusion of cognitively healthy individuals only, but it is also possible that individuals with more severe T2DM pathophysiology or poorly controlled T2DM were underrepresented at follow‐up, potentially leading to an underestimation of the true associations. In concordance, glycemic control was generally good in our cohort, with 95% of participants showing well‐controlled HbA1c values. This may further limit the generalizability of our findings to populations with poorer metabolic control. However, our results indicate a biologically plausible connection between T2DM and the development of MCI 5 years later, as the subgroup with the poorest glycemic control—middle‐aged men—were also at the greatest risk of MCI. We did not include data on the duration of T2DM, which has been reported to influence the development of dementia and may also be associated with MCI.^43^ However, we expanded our analyses by including data on T2DM at t0 (10 years prior to MCI diagnosis), and the results remained robust, showing an even stronger association between T2DM and naMCI in both the overall population and among middle‐aged men. Our data lack biomarker information to distinguish between different underlying pathologies for incident MCI. In additionally, due to the small number of cases, we did not apply the subjective cognitive decline criteria for diagnosing MCI. However, we conducted a sensitivity analysis to address this, which showed robust effects for men and weakened effects for women.

Our study has several strengths. We analyzed a large, well‐characterized, population‐based prospective cohort study, allowing us to include only cognitively unimpaired participants at t1 and track incident MCI cases five years later. The cognitive assessment conducted at t1, which demonstrated high accuracy in identifying MCI cases in a previous study,^21^ facilitates reliable identification of incident MCI cases. Furthermore, we excluded participants with previously undiagnosed T2DM to minimize the likelihood of misclassifying non‐diabetic individuals as diabetic.

In summary, T2DM in cognitively unimpaired participants was associated with an increased risk of incident MCI after five years, especially for naMCI in middle‐aged men. Thus our analyses confirm T2DM as a risk factor for incident MCI. These results underscore the importance of enhancing sex‐specific tailored prevention efforts at the population level to prevent T2DM and preserve cognitive performance. Furthermore, for individuals in whom T2DM has developed, intervention studies must demonstrate whether improved glycemic control can reduce the risk of MCI. Addressing both the prevention of T2DM and the management of blood glucose levels in affected individuals is crucial for maintaining cognitive health and enhancing overall quality of life.

## CONFLICT OF INTEREST STATEMENT

All authors declare that they have no competing interests or conflicts. Author disclosures are available in the Supporting Information.

## CONSENT STATEMENT

The authors confirm that all participants of the study provided written informed consent.

## Supporting information



Supporting Information

Supporting Information

Supporting Information
