# Supplementary material for: Sex‐ and age‐specific effect of known type 2 diabetes mellitus on incident mild cognitive impairment five years later: Results from the population‐based Heinz Nixdorf Recall study
Source: Alzheimers Dement (Amst). 2025 Jun 11;17(2):e70130. doi: 10.1002/dad2.70130 (PMC12152370; doi:10.1002/dad2.70130)
Supplement: Supplementary file 1 — Supporting Information [file DAD2-17-e70130-s001.pdf]

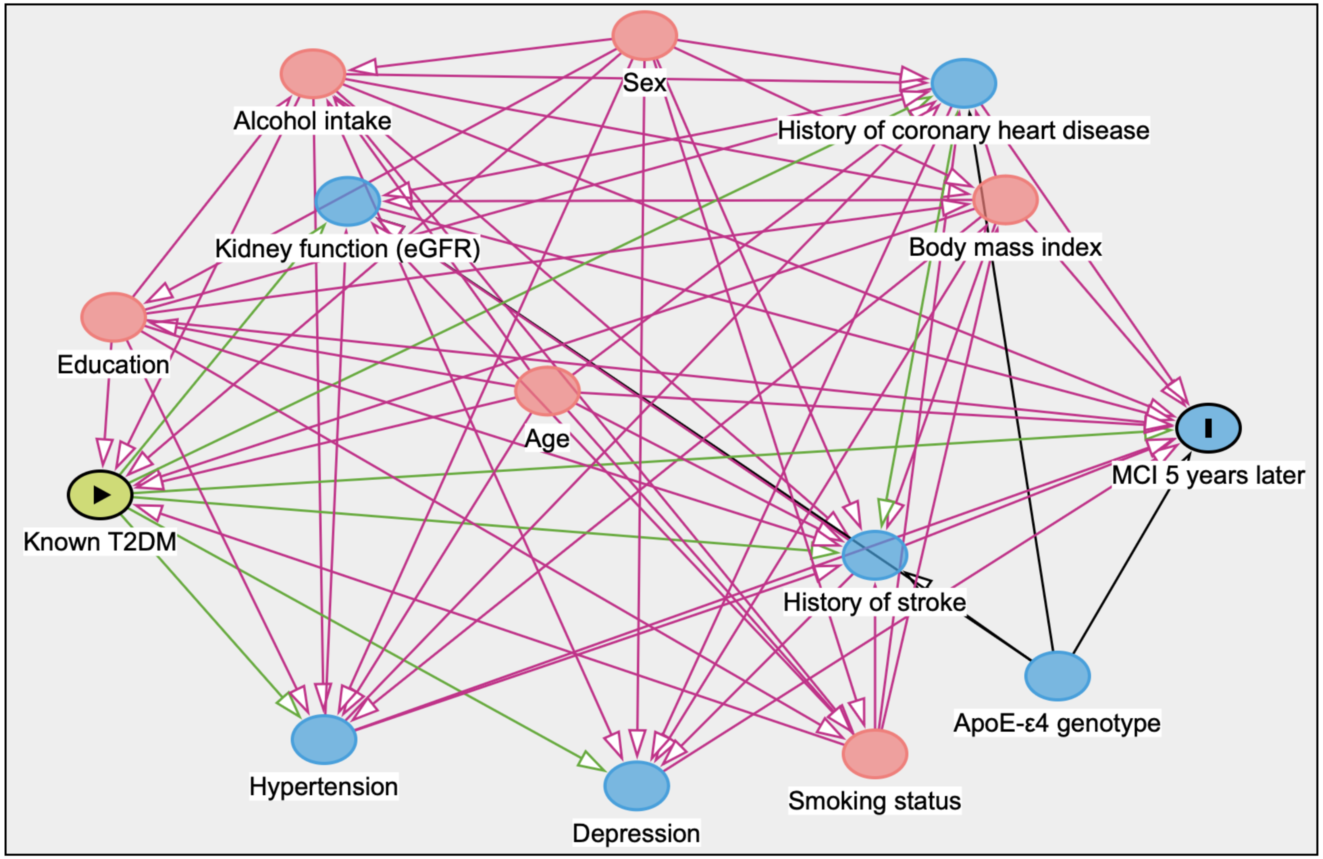

SUPPLEMENTARY FIGURE 1

Directed acyclic graph (DAG) used to determine the confounding covariates for the regression analyses adjustment set of this study.

The exposure of interest is marked in green (known type2 diabetes mellitus (T2DM) at t1); the outcome of interest is marked in blue with an "**I**" (Mild cognitive impairment (MCI) five years later). The ancestor of outcome are also marked in blue. The ancestors of exposure and outcome are marked in red. The minimally sufficient adjustment set included the following variables: age, sex (only for the total sample), education, body mass index, smoking status, and alcohol intake. Based on this framework, hypertension, depression, history of coronary heart disease, Apolipoprotein E ε4 genotype (AoeE- ε4), history of stroke and kidney function (estimated glomerular filtration rate (eGFR)) were not included in the main adjusted models.The DAG was constructed using the online software DAGity v2.3 [28].
