# Supplementary material for: Sex‐ and age‐specific effect of known type 2 diabetes mellitus on incident mild cognitive impairment five years later: Results from the population‐based Heinz Nixdorf Recall study
Source: Alzheimers Dement (Amst). 2025 Jun 11;17(2):e70130. doi: 10.1002/dad2.70130 (PMC12152370; doi:10.1002/dad2.70130)
Supplement: Supplementary file 2 — Supporting Information [file DAD2-17-e70130-s003.docx]

**Supplementary Table 1.** Comparison of sociodemographic, clinical and cognitive characteristics assessed at t1 between the participants included in the study (*n*=1467) and participants who did not participate in the follow-up examination (t2; non-returners, *n*=813) as well as with those who participated in the follow-up examination but were excluded from the analysis due to our exclusion criteria (drop-outs, *n*=1518); Heinz Nixdorf Recall Study, Germany, 2005-2008 (t1) and 2010-2015 (t2).

|  | Participants included (1) | Non-returners (2) | Drop-outs (3) | p value (1, 2) ^***^ | p value (1, 3) ^***^ |
| --- | --- | --- | --- | --- | --- |
| Number of participants | 1467 | 813 | 1518 |  |  |
| Age, mean ±SD | 62,8 (7,1) | 66,4 (8,0) | 64,0 (7,5) | <.001 | <.001 |
| Education |  | ^¶^ | ^‡‡^ | <.001 | <.001 |
| ≤ 10 years, n (%) | 106 (7) | 125 (15) | 136 (9) |  |  |
| 11-13 years, n (%) | 778 (53) | 463 (57) | 868 (57) |  |  |
| 14-17 years, n (%) | 333 (23) | 159 (20) | 375 (25) |  |  |
| ≥18 years, n (%) | 250 (17) | 65 (8) | 135 (9) |  |  |
| BMI, kg/m^2^, mean ±SD | 27,7 (4,5) ^*^ | 28,8 (5,2) | 28,3 (4,7) | <.001 | <.001 |
| Smoking |  | ^#^ | ^§§^ | <.001 | .474 |
| Never smoked, n (%) | 632 (43) | 316 (39) | 662 (44) |  |  |
| Former smoker, n (%) | 604 (41) | 313 (39) | 617 (41) |  |  |
| Current smoker, n (%) | 231 (16) | 180 (22) | 237 (16) |  |  |
| Alcohol, g/day, mean ±SD | 10,3 (15,2) ^†^ | 7,0 (12,7) | 9,2 (14,8) | <.001 | .018 |
| Known T2DM, n (%) | 63 (4) | 86 (11) | 115 (8) |  |  |
| Glucose, mg/dl, mean ±SD | 107,3 (17,9) | 117,7 (34,1) | 114 ,0 (26,9) | <.001 | <.001 |
| HbA1c, %, mean ±SD | 5,50 ±0,56 ^§^ | 5,9 (1,0) ^**^ | 5,7 (0,8) ^¶¶^ | <.001 | <.001 |
| HbA1c ≥ 6,5 %, n (%) | 72 (5) ^§^ | 123 (15) ^**^ | 140 (9) ^¶¶^ | <.001 | <.001 |
| Word list immediate recall, number of recalled words, mean ±SD | 5,9 (1) | 4,7 (1,4) | 4,9 (1,3) | <.001 | <.001 |
| Word list delayed recall, number of recalled words, mean ±SD | 4,3 (1,5) | 2,8 (1,8) | 2,7 (1,7) | <.001 | <.001 |
| Labyrinth test, seconds, mean ±SD | 42,6 (16,8) | 66,8 (41,35) | 64,8 (41,1) | <.001 | <.001 |
| Verbal fluency test, number of named animals, mean ±SD | 25,4 (5,6) | 20,8 (6,5) | 20,8 (6,0) | <.001 | <.001 |
| Clock Drawing Test, score ≥3; n(%) | 0 (0) | 238 (29,3) ^††^ | 589 (38,9) ^##^ | <.001 | <.001 |

Abbreviations: BMI = body mass index (kg/m^2^); MCI = Mild cognitive impairment defined following previously published criteria excluding subjective cognitive decline, SD = standard deviation, t1 = first follow-up (2005-2008), t2 = second follow-up (2010-2015); Please note that we have only include cognitively healthy participants at t1 in our analyses to examine incident MCI risk in the further course.

^*^missing values for *n*=5 participants

^†^missing values for *n*=11 participants

^‡^ missing values for *n*=9 participants

^§^ missing values for *n*=22 participants

^¶^ missing values für *n*=1 participants

^#^ missing values für *n*=4 participants

^**^ missing values für *n*=22 participants

^††^ missing values für *n*=26 participants

^‡‡^ missing values für *n*=4 participants

^§§^ missing values für *n*=2 participants

^¶¶^ missing values für *n*=33 participants

^##^missing values für *n*=3 participants

^***^ Comparisons between groups calculated using t-tests, Mann-Whitney U test or Pearson χ2-test as appropriate
